# Clustered Data Muling in the Internet of Things in Motion†

**DOI:** 10.3390/s19030484

**Published:** 2019-01-24

**Authors:** Emmanuel Tuyishimire, Antoine Bagula, Adiel Ismail

**Affiliations:** ISAT Laboratory, University of the Western Cape, Cape Town, Bellville 3575, South Africa

**Keywords:** clustering, hybrid network, UAV

## Abstract

This paper considers a case where an Unmanned Aerial Vehicle (UAV) is used to monitor an area of interest. The UAV is assisted by a Sensor Network (SN), which is deployed in the area such as a smart city or smart village. The area being monitored has a reasonable size and hence may contain many sensors for efficient and accurate data collection. In this case, it would be expensive for one UAV to visit all the sensors; hence the need to partition the ground network into an optimum number of clusters with the objective of having the UAV visit only cluster heads (fewer sensors). In such a setting, the sensor readings (sensor data) would be sent to cluster heads where they are collected by the UAV upon its arrival. This paper proposes a clustering scheme that optimizes not only the sensor network energy usage, but also the energy used by the UAV to cover the area of interest. The computation of the number of optimal clusters in a dense and uniformly-distributed sensor network is proposed to complement the k-means clustering algorithm when used as a network engineering technique in hybrid UAV/terrestrial networks. Furthermore, for general networks, an efficient clustering model that caters for both orphan nodes and multi-layer optimization is proposed and analyzed through simulations using the city of Cape Town in South Africa as a smart city hybrid network engineering use-case.

## 1. Introduction

The use of Unmanned Aerial Vehicles (UAVs) continues to be not only one of the most efficient approaches, but also less expensive and risky ones, for various exploratory problems. These problems include rescuing, data delivery/collection, surveillance, and many more. In the case of city surveillance, it has been found efficient to assist UAVs with a Sensor Network (SN) comprising static ground sensors, which collect local information and deliver them to the UAVs visiting them [1]. In case more detailed information is to be captured, large-scale and complex SNs are usually deployed in the zone of interest. In this case, the use of UAVs continues to be one of the most efficient ways to handle the mentioned situations. However, UAVs’ flights are generally constrained by their limited flight time, fuel and energy usage when powered by battery. Therefore, the UAV exploration of targeted environments necessitates the optimization of energy usage to ensure the scalability and resilience of the data capturing. This is why it is generally important to minimize the UAVs’ moves, yet collect maximal information by having the UAVs visit only an optimal number of selected ground sensors serving as ground gateways, each of them receiving information collected from other ground sensor nodes for collection and data muling by a UAV upon its visit. It is then important to assign to each gateway an optimal team of sensor nodes providing the sensed data. Here, we refer to the teams of sensor nodes as cluster members, while their gateways are referred to as cluster heads, and the corresponding partition of the sensor network is called clustering.

Cluster-based sensor networking has been a subject of high interest in the literature. In [2], the physical-access control cross-layer analytical approach for determining the optimum number of clusters has been proposed. The proposed model minimizes the communication-energy consumption in a highly-dense sensor network. In [3], the Euclidean distance (communication range and the area on which the network is deployed) from nodes to a cluster head was considered in order to design clusters with the objective of minimizing the energy required for efficient communication. In the latter paper, the energy usage is minimized with the increase of the number of clusters. A connectivity-based k-hop to the cluster head was proposed as a clustering technique in [4], where it was shown that the efficiency of messages transmissions from the cluster heads to the sink of the underlying network is reduced with the number of clusters. This raises the issue of finding the optimal number of clusters in a network (note that it exists). An optimal, temporal clustering algorithm was proposed in [5], as an adaptive model for a wireless micro-sensor network, to ensure efficient utilization of its energy. In [6], the optimal number of cluster heads and their locations were analytically computed for efficient wireless sensor network communication. The main goal of the paper was to ensure optimal data transfer in the network by adopting the cluster head selection method in [7], which is based on the calculated probability of a node to be a cluster head. Simulations in [6] showed a better performing clustering, compared to the *k*-means algorithm-based [8] schemes, including those presented in [9,10,11,12,13].

The k-means is a clustering algorithm aiming to partition nodes into Voronoi cells (see [14] for example). Given the number *k* of centroids (cluster heads), the algorithm consists of the following steps.

1. Initialization: this is done by randomly selecting *k* of the nodes to be cluster heads.
2. Assignment step: this step consists of assigning cluster members to cluster heads, based on the least Euclidean distance between the node and cluster heads.
3. Update: for each cluster, a centroid (most central node) is computed, and if it is different from the current cluster head, it replaces it.
4. Iteration: this step consists of alternating Steps 2 and 3 until no more updates are possible.

The clustering problem being an NP-hard problem, the k-means is its heuristic solution, whose properties include: (i) local convergence, (ii) the choice of the number *k* of cluster heads influencing the optimality of the clustering, (iii) the initialization step impacting the running time of the algorithm, and (iv) Euclidean distance used as the utility function for clustering. To address issues related to the above four properties, different versions of the algorithm have been proposed, respectively a globally-converging clustering [15], an optimal number of clusters for image segmentation [16,17], a better initialized k-means [18] algorithm, and the multi-norm clustering [19]. However, to the best of our knowledge, there is no k-means algorithm that has been proposed to ensure the connectivity of all cluster members to corresponding cluster heads in order to avoid orphan/isolated nodes in a sensor network. Two versions of the k-means algorithm were considered in [6]: (i) the deterministic k-means algorithm, which is built around the same principles as the classical k-means algorithm, and (ii) the adaptive k-means algorithm, which uses the classical k-means algorithm iteratively to cluster *n* sensor nodes for *n* times by varying the parameter *k* from 1 to *n* and selecting the clustering result with the minimum energy cost. The Distance-based Crowdedness Clustering (DCC) was also proposed in [6] as a greedy algorithm, which, for a given general network, outputs the corresponding clustering by using node degrees as a way of selecting the best cluster head (one of highest degree) and building corresponding clusters. In DCC, the length of the underlying network’s links is used to select the clustering radius (the length of one of the links), and every neighbor of the selected cluster head at a distance less than the radius is added to the cluster. This process continues to all remaining nodes, until each node belongs to a cluster. After performing clustering once, the corresponding cost (a function of the radius) is computed, and for all possible values of the radius, a clustering corresponding to the least cost is chosen to be the output of the algorithm.

**Figure 1:**
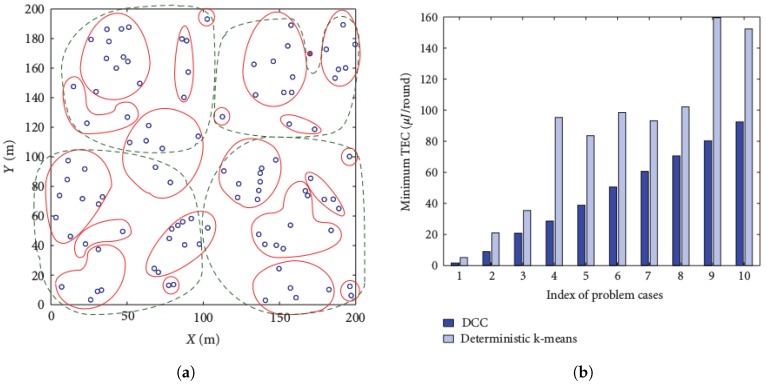
Comparison between Distance-based Crowdedness Clustering (DCC) and deterministic k-means [6]. (**a**) Clustered nodes; (**b**) minimum energy per round. TEC, Total Energy Consumption.

Figure 1a compares DCC and the adaptive k-means algorithms. In the figure, the solid lines represent clusters with the DCC, while the dotted lines represent the adaptive k-means clustering for 100 nodes, on a 200 m × 200 m area. The figure reveals that DCC outperforms k-means in terms of cluster-based node density. On the other hand, Figure 1b shows that the DCC outperforms the k-means in terms of Total Energy Consumption (TEC) efficiency for 10 consecutive runs of both algorithms.

While the models and algorithms presented above focused the optimization process on a ground-based/terrestrial sensor network, the works in [20,21,22,23,24] are among those that have addressed UAVs’ related clustering. In [20], UAVs were presented as moving agents, which were clustered by using their mobility attributes to predict their motion, hence leading to clusters’ predictions. In [21], the UAVs also were clustered with the goal of computing the optimal route discovery. In [22], a clustering scheme was proposed to provide Internet connectivity, using a mobile sink (a UAV). In the paper, the clustering was performed based on the distance separating potential cluster heads and other ground sensor nodes, and also the proximity of the UAV, and a UAV’s move was predicted in order to determine its corresponding cluster. To the best of our knowledge, this was one of the first clustering schemes considering different positions of a UAV (path). However, the paper did not consider the energy spent by the UAV while moving from one position to another, which is a requirement to allow the UAV to aggregate data as much as possible prior to being recharged. The works in [23,24] considered a multi-layer model with a team of UAVs playing the role of an airborne gateway network for a terrestrial sensor network. While [23] proposed an initial model showing through simulation how the multi-layer network can be designed, the model in [24] was based on MIMO clusters to increase the terrestrial sensor network lifetime by avoiding disconnections that can lead to orphan/isolated sensors or groups of sensors that are unable to deliver their data.

### Motivation and Contribution

As discussed in Section 1, the k-means algorithm (see [8] for example) and its variants are some of the most popular clustering models. This algorithm aims to minimize the sum of distances (standard deviations) between *k* cluster heads and their cluster mates. The deterministic k-means algorithm finds the best number *k*, and a clustering cost function may be used to evaluate the cost corresponding to each value of *k* ranging from one to the number of observations. Alternatively, mathematical methods using calculus are used to compute the number *k*. When the connection (affinity) between cluster members is one of the requirements, this algorithm is outperformed in terms of TEC, even in dense networks (see [6]). Note that for the k-means algorithm, nodes are grouped based on their statistical characteristics. However, statistical approaches alone could be less efficient in case the relationship of observations matters. The affinity of data points/nodes has been addressed in [25,26,27], but could not guarantee a perfect assignment of the node to the correct cluster head (the node to which all cluster members are connected). This is why the DCC algorithm proposed in [6] could outperform the adaptive k-means algorithm.

This paper extends [28] to revisit the problem of clustering as a way of optimizing hybrid terrestrial/airborne sensor networks by proposing a novel clustering model that combines efficient sensor network communication and efficient cluster heads’ visitation by a UAV. The clustering problem for a hybrid network (UAV routes and the communication-based SN) is firstly proposed. Thereafter, the optimal number of clusters is rigorously computed for uniform and dense network distribution settings. A heuristic clustering is then proposed for general networks; and its extension to cater for the sensor nodes’ isolation is supported through relaxation techniques. Our work is closely related to DCC in [6], but differs by proposing a clustering scheme that (i) takes care of the relationship of nodes while DCC does not and (ii) considers a multi-layer approach that caters for the efficient cluster heads’ visitation by a UAV. While different clustering schemes and algorithms have been proposed in the literature, they have either focused the optimization process on a single layer (UAV layer or terrestrial layer) [2,3,6,8,9,10,11,12,13] or consisted of non-optimization techniques that show how UAVs can be used as mobile sensor networks [1] for different purposes including city surveillance. Our model is based on an optimization process that considers both layers of a hybrid sensor network. Furthermore, the presence of orphan nodes (which could be either cluster heads or normal nodes) may lead to (i) a dislocated network with part of the data produced by the orphan nodes not reaching the network gateway and (ii) an energy-inefficient hybrid network with a UAV’s energy being wasted to visit an orphan cluster head that does not have data to be collected. While all previous works have discounted the issue of orphan nodes, the clustering solution presented in this paper addresses this issue by the proposed mitigation processes to reduce the number of orphan nodes.

The rest of the paper is organized as follows. The problem is mathematically formulated in Section 2, and the proposed algorithmic solution is described in Section 3. To adopt a special case, the proposed algorithm is relaxed in Section 4, and the performance of the proposed model is discussed in Section 5, whereas in Section 6, the paper is concluded.

## 2. Problem Formulation

**Figure 2:**
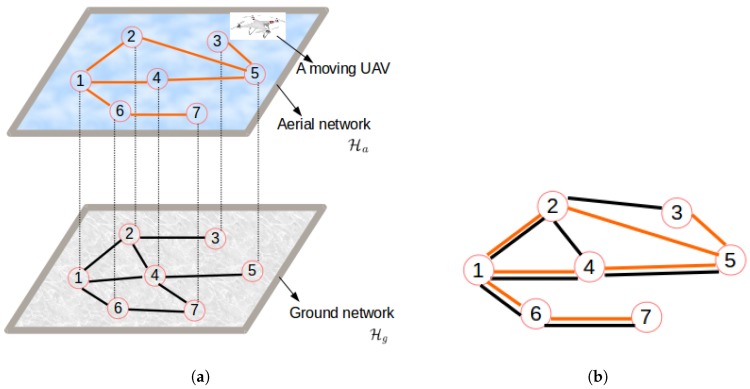
Terrestrial, airborne, and hybrid networks. (**a**) Physical topology; (**b**) conceptualized topology.

In this section, the clustering problem is formalized as an energy optimization problem, under network-related constraints. The focus lies on an energy-efficient design where a single UAV located at a specific base station is used to collect sensor data from a number of collection points. The network H can be considered as a hybrid network $H(H_{g},H_{a})$ combining the terrestrial sensor sub-network $H_{g}$ and the airborne muling sub-network $H_{a}$ consisting of all possible UAVs’ paths. Note that while having the same number of nodes, the $H_{a}$ network might differ from $H_{g}$ as it is based on potential UAV path restrictions related to obstacles and Distance-based Crowdedness Clustering (DCC) different environmental limitations. This is illustrated by Figure 2.

Figure 2 reveals that while the two network configurations in Figure 2a (aerial and ground networks) have the same sets of nodes, they may have different sets of links and hence different routing paths. Therefore, they may result in different energy consumption patterns ($E_{g}\neq E_{a}$). This raises the issue of energy consumption in a hybrid network (Figure 2b) and the need for an optimization model that combines the energy consumed by both networks $E_{h}=f\left(E_{g},E_{a}\right)$.

### 2.1. The Energy Models

As suggested earlier, this paper considers an energy-efficient model where the energy consumption is described below.

(1) $$\begin{array}{ccc} E_{g} & = & E_{t}+E_{r} \end{array}$$

(2) $$\begin{array}{ccc} E_{a} & = & \beta E_{c}+\gamma E_{u}, \end{array}$$

where the constants β and γ are proportionality constants corresponding to $E_{c}$ and $E_{u}$, respectively, and the energy components $E_{r}$, $E_{t}$, $E_{c}$, and $E_{u}$ are defined below.

- Energy for sensor-data reception ($E_{r}$): This is the energy spent by cluster heads due to its topological and environmental properties, the physical/electronic properties of the receiving node, and the nature of messages to be received. We assume that all possible cluster heads are in the same and good condition; hence, they require the same quantity of energy to receive a message. It is assumed that nodes communicate directly with their corresponding cluster head, and in the case a multi-hop communication is applicable, the least interference beaconing protocol (see [29]) is used to find sensor communication route.
- Energy for data transmission among sensors ($E_{t}$): This is the total energy required to move the captured data from each cluster node to its corresponding cluster head. This form of energy is directly proportional to the distance separating the two communicating sensors. We assume one-hop inter-cluster communication, and hence, the considered distance is the Euclidean length of links. All nodes of the network are assumed to require the same quantity of energy for message transmissions.
- Energy for UAV data transport ($E_{u}$): This refers to the expected energy required for a UAV to visit cluster heads. This energy depends on the number of cluster heads in the $H_{g}$ network and the distance between these nodes (the expected link length).
- Energy for UAV data collection ($E_{c}$): This is the energy spent by the UAV to collect data from the sensor nodes (cluster heads).

From Equation (1), the overall energy for data dissemination in the terrestrial ground-based sensor network to cluster heads and data muling by the UAV can be expressed by the weighted sum of energy consumption in both ground and airborne networks as expressed by:

(3) $$E_{h}=\alpha E_{g}+\beta E_{u}+\gamma E_{c}.$$

#### 2.1.1. The Terrestrial Network Energy Consumption: $E_{g}$

Let L×L units of area be the area of the field where sensors are distributed. It follows that one cluster’s area is $\sqrt{L^{2}/k}\times \sqrt{L^{2}/k}$, based on the Voronoi diagram (see [30]).

It has been shown in [6] that the total energy E for data gathering in a uniformly-distributed network of type $H_{g}$ is expressed as follows.

(4) $$E=\left(2n-2k+a\times k\right)E_{e}+nE_{p}+\left(n-k\right)e_{f}\frac{L^{2}}{3k}+a\times k\times e_{m}\frac{4L^{4}}{9},$$

where *a* (with 0<a≤1) denotes the data compression ratio: an input of *k* bits results in an output of a×k bits after compression; $E_{e}$ denotes the energy for driving the electronics; $E_{p}$ is the energy for data processing; *n* the number of all sensors in the field; and the constants $e_{f}$ and $e_{m}$ represent the coefficient corresponding to the effects of the clusters intra-distances and inter-distances, respectively.

The considered case in this paper assumes that there is no inter-cluster communication, and thus,

$$e_{m}=0.$$

This is why the gathering energy $E_{g}$ for the uniformly-distributed network of type $H_{g}$ is computed as follows.

(5) $$E_{g}=\left(2n-2k+a\times k\right)E_{e}+nE_{p}+\left(n-k\right)e_{f}\frac{L^{2}}{3k},$$

On the other hand, for the generally-distributed network, the energy may be computed as follows. Let C be a set of clusters; $c_{i}$ represents the node *i* of cluster *c*, and $c^{h}$ denotes the cluster head of cluster *c*.

(6) $$E_{g}=\left(2n-2k+a\times k\right)E_{e}+nE_{p}+\sum_{c\in C}\sum_{i\in c}d\left(c_{i},c^{h}\right),$$

where the function $d(c_{i},c^{h})$ represents the Euclidean distance between node *i* and the cluster head in the cluster *c*.

#### 2.1.2. The Data Collection Energy Consumption: $E_{c}$

This is the total energy for data collection from cluster heads by a UAV. Let 1, 2, …*k* be the indices corresponding to *k* cluster heads. If $E_{i}$ is the energy required by the UAV to receive data from the cluster head *i* (with 1≤i≤k) and $e_{i}$ is the energy required by the cluster head to forward the gathered data to the UAV, then the total energy $E_{c}$ for data collection is expressed as follows.

(7) $$E_{c}=\sum_{i=1}^{k}\left(E_{i}+e_{i}\right)=\frac{k}{k}\sum_{i=1}^{k}\left(E_{i}+e_{i}\right)$$

Hence,

(8) $$E_{c}=k\left(\bar{E}+\bar{e}\right),$$

where $\bar{E}$ and $\bar{e}$ are the expected value of the energy required to receive and forward data, respectively.

#### 2.1.3. Energy for UAV Transportation: $E_{t}$

The transportation energy $E_{t}$ depends on the length of the used path, which gets longer as the number of clusters increases. We assume that the UAV moves from one node to another, using Dijkstra’s algorithm [31] on the network of type $H_{a}$. This will enable us to evaluate the goodness of a node to be in a particular cluster or even to be a cluster head.

Since the UAV-transportation energy is directly proportional to the length of the path used, it is directly proportional to the number of cluster heads. It is also proportional to the average distance from one cluster head to another and hence the distance *D* to travel from one node to another. Therefore, $E_{t}$ is computed as follows,

(9) $$E_{t}=b\times k\times D.$$

where *b* is the proportionality constant and $D=E(E_{j}\left(d\right))$ is the expected value of the average length of shortest paths *d* from each sensor node *j* to others, where $E_{j}\left(d\right))$ expresses the expected Dijkstra’s shortest distance *d* from the node *j* to any node in the underlying network (here, it is $H_{a}$).

Considering a network whose number of nodes is *n*, let $A_{n\times n}=\left\{d_{ij}\right\}$ be the matrix where each entry $d_{ij}$ corresponds to the shortest distance from node *i* to node *j* based on Dijkstra’s algorithm. Here, $d_{ii}=0\;\forall i$ because $d_{ii}$ represents the distance from node *i* to itself. The index *D* may be calculated as follows.

$$D=\frac{1}{n-1}\sum_{j=1}^{n}\left(\frac{1}{n-1}\sum_{i=1}^{n}a_{ij}\right)=\frac{1}{{\left(n-1\right)}^{2}}\sum_{j=1}^{n}\sum_{i=1}^{n}a_{ij}$$

Notice that the denominator is n−1 to exclude the case where i=j with related terms equal to zero.

It follows from Equations (3), (5), (8) and (9) that the total energy used in data collection is expressed as follows.

(10) $$E_{h}\left(k\right)=E_{g}+bkD+k\left(\bar{E}+\bar{e}\right).$$

The main issues involved in the optimal clustering model considered in this work are (i) finding the optimal number of clusters, (ii) selection of the optimal cluster heads/sinks, and (iii) associating the cluster members with the sinks. These issues can be solved by three algorithmic solutions: (a) a myopic k-means clustering algorithm where the optimal number of clusters $k=K_{opt}$ is computed and the classical K-means algorithm is applied with $k=K_{opt}$, (b) an optimized k-means clustering algorithm where the optimal number of clusters $k=K_{opt}$ is computed and the $K_{opt}$ best cluster heads are selected and fed to the k-means algorithm to guide the clustering process, and (c) a multi-step clustering algorithm where a sequence of cluster head selection and cluster member association is performed on the network until all the nodes are assigned a cluster head or member status. Note that while the k-means algorithm can be applied to a dense and uniform network where each sensor node is able to communicate with its neighbors, the multi-step algorithm is more suitable for general networks where the connectivity property may not be met.

### 2.2. Problem Definition

The network considered in this paper is denoted by $H(N,P_{g},P_{a},E_{g},E_{a})$, where N is the set of sensor nodes and the UAVs’ base stations’ locations, $P_{g}$ is the set of paths expressing possible sensors communication pathways in the ground-based terrestrial network, $P_{a}$ the set of paths in the airborne network consisting of possible routes followed by the UAVs to collect data delivered by the ground-based sensor network, and the energy consumed by the set of paths $P_{g}$ and $P_{a}$ is respectively represented by $E_{g}$ and $E_{a}$. Given a hybrid network H, the problem consists of finding the smallest nodes’ partition P(N) to minimize the total energy $E_{h}$ (see Equation (3)), such that each partition (cluster) is connected and its optimum head is known. The energy $E_{h}$ is referred to as the clustering cost. The network design consists of finding a network configuration that minimizes the clustering cost function subject to node selection and topology constraints with the objective of partitioning the network into two sets: a dominating set of UAV collection points and a dominated set of cluster members forming the edge of the network. Mathematically formulated, the design process consists of finding a network partition *C* derived from the graph of the type explained in Figure 2b, which leads to the optimal energy consumption $E_{opt}$, such that N is divided into disjoint clusters, where the cluster head is communicatively connected with all its cluster mates.

(11a) $$\begin{array}{cc} E_{opt} & =\min E_{h}=\min\left(\alpha E_{g}+\beta E_{u}+\gamma E_{c}\right) \end{array}$$

Subject to,

(11b) $$\forall c\in C,\;\exists x\in c,\forall y\in c,\left(x,y\right)\in P_{g}$$

(11c) $$\;c_{1},c_{2}\in C,c_{1}\cap c_{2}=\emptyset$$

(11d) $$\underset{c\in C}{⋃}c=N$$

where, Constraints (11b) shows the dominating set property of the set of cluster heads and (11c) and (11d) represent the network partitioning properties.

## 3. The Proposed Clustering Models

Two clustering algorithms were developed:

1. The UAV-Aware k-Means (UAKM) algorithm, which computes the number *k* of optimal clusters for hybrid dense networks to support/complement k-means clustering. Here, the number *k* is calculated using both the ground and the aerial networks, and hence, it considers the movement of the UAV.
2. The UAV-Aware DCC (UADC) algorithm, which adapts the DCC algorithm to include the UAVs data collection process.

### 3.1. The UAV-Aware K-Means Algorithm

In this subsection, we express the forms of energy in terms of the number of clusters *k* a hybrid network (see Section 2.2) needs to be partitioned into and use calculus to compute the value *k* that minimizes the total energy required for data collection. Energy Equation (10) can be expressed in terms of the number of clusters k, which in turn can be used to determine the optimal number of clusters, as shown in Equation (12).

(12) $$\frac{\partial E}{\partial k}=E_{e}\left(a-2\right)+\frac{L^{2}e_{f}\left(k-n\right)}{3\;k^{2}}-\frac{L^{2}e_{f}}{3\;k}+bD+\bar{E}+\bar{e}$$

By solving the equation, $\frac{\partial E_{h}}{\partial k}=0$, where k≥1, we obtain the optimal value of $E_{h}$ for:

(13) $$K_{opt}=\sqrt{\frac{\;L^{2}e_{f}n}{3(\;E_{e}\left(a-2\right)+\;bD+\bar{E}+\bar{e})}}$$

Notice that the second derivative is:

(14) $$\frac{\partial^{2}E_{h}}{\partial k^{2}}=-\frac{2\;L^{2}e_{f}\left(k-n\right)}{3\;k^{3}}+\frac{2\;L^{2}e_{f}}{3\;k^{2}}.$$

We know that all observables in Equation (14) are positively valued. Furthermore, the difference k−n is always negative (the number of cluster heads cannot exceed the number of all existing nodes). It follows that,

(15) $$\frac{\partial^{2}E_{h}}{\partial k^{2}}\geq 0.$$

This confirms that the the total energy $E_{h}\left(k\right)$ is a minimum at $K_{opt}$, as shown in Equation (13).

Since the optimal number of clusters has to be a positive integer, the optimal number of clusters is denoted by K, and it is calculated as follows.

(16) $$K_{opt}=\left\{\begin{array}{cc} \left\lceil k\right\rceil & \;\mathrm{if}\;E(\lceil k\rceil )\leq E(\lfloor k\rfloor )\\ \left\lfloor k\right\rfloor & \mathrm{otherwise} \end{array}\right.$$

**Figure 3:**
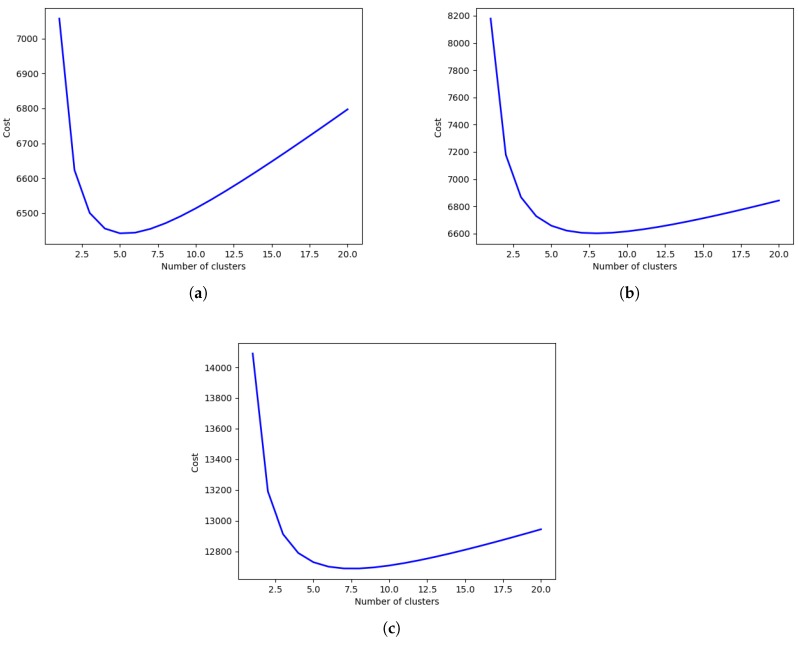
Energy required versus the number of clusters: (**a**) 30 m × 30 m network with 100 nodes; (**b**) 60 m × 60 m network with 100 nodes; (**c**) 30 m × 30 m network with 200 nodes.

Consider three networks on which the following parameters are defined in Table 1. The corresponding graphs of the energy are shown in Figure 3.

In Table 1, $\bar{E}+\bar{e}$ can be set to zero if we need to consider a case where some sensors are located together with refueling/repairing stations, which increase the energy of a UAV even if it were collecting data.

Figure 3, showing the energy required compared to the number of clusters, reveals the optimal number of clusters for the three different networks. Figure 3a–c shows that the optimal number of clusters increases with the network size. Figure 3a shows that the number $K_{opt}$ for the first network (Network 1) lies in the interval (4,5). On the other hand, using Equation (13), $K_{opt}=4.84$. It follows from Equation (16) that,

(17) $$K_{opt}=\left\{\begin{array}{cc} 5 & \;\mathrm{if}\;E(5)\leq E(4)\\ 4 & \;\mathrm{otherwise}. \end{array}\right.$$

Thus, the optimal number of clusters in this case is $K_{opt}=5$. Similarly, it can be shown that for the second network (Network 2),

(18) $$K_{opt}=\left\{\begin{array}{cc} 6 & \;\mathrm{if}\;E(6)\leq E(5)\\ 5 & \;\mathrm{otherwise}. \end{array}\right.,$$

leading to $K_{opt}=6$. For the third network (Network 3),

(19) $$K_{opt}=\left\{\begin{array}{cc} 7 & \;\mathrm{if}\;E(7)\leq E(6)\\ 6 & \;\mathrm{otherwise}. \end{array}\right.,$$

leading to a value of $K_{opt}=7$.

### 3.2. The UAV-Aware DCC Algorithm

The UADC algorithm has been designed based on a multi-step process using the following cluster head selection assumptions:

- Degree-aware selection policy, where nodes are assigned the cluster head identity based on their node degree deg(i). While leading to the UAV choosing data collection points with a high volume of data, this policy might lead to the UAV flying longer distances to collect these data, and hence, depleting its energy during its inbound journey.
- Distance-aware selection policy, where nodes are elected cluster heads based on the expected Dijkstra’s shortest path from the nodes to all other nodes, following the links in the airborne network (links of the network $H_{a}$). This policy aims at minimizing the energy usage of the airborne sensor network, but might lead to the UAV being tasked with collecting data at collection points with very few data.
- A hybrid policy that combines features from dense and distance-aware cluster head selection by combining both parameters into a weighted sum metric expressed by:
  (20) $$P\left(i\right)=\lambda deg\left(i\right)+\psi \frac{1}{D_{i}}.$$

Here, deg(i) represents the number of available neighbors (of node *i*) in the network of type $H_{a}$, whereas $D_{i}$ is the average distance from node *i* to all nodes in the network of type $H_{g}$. λ and ψ are coefficients corresponding to the node degree in $H_{g}$ and average distance in $H_{a}$, respectively.

This policy is used in clustering as shown by the proposed algorithm described as follows.

**Input:** The graph of type $H(H_{a},H_{g})$
**Output:** A dictionary of cluster heads and their cluster mates

In Algorithm 1, the first steps consist of computing a list *L* of all link lengths in the SN (network of type $H_{g}$) and the dictionary $D_{P}$, whose keys are the sensor labels, and the corresponding values consist of the average distance to each node in the restricted network (network of type $H_{a}$). The minimum coverage energy $E_{min}$ is initialized to infinity. The network clustering is expressed in the form of a dictionary whose keys are the cluster heads, and the values correspond to the clusters’ members. The clusters’ dictionary *C* is initially set to empty (Line 4). The cluster dictionary is assumed to have the cluster heads as keys, and their corresponding values are the list of nodes each cluster head is to support.

**Algorithm 1:** Optimal clustering.

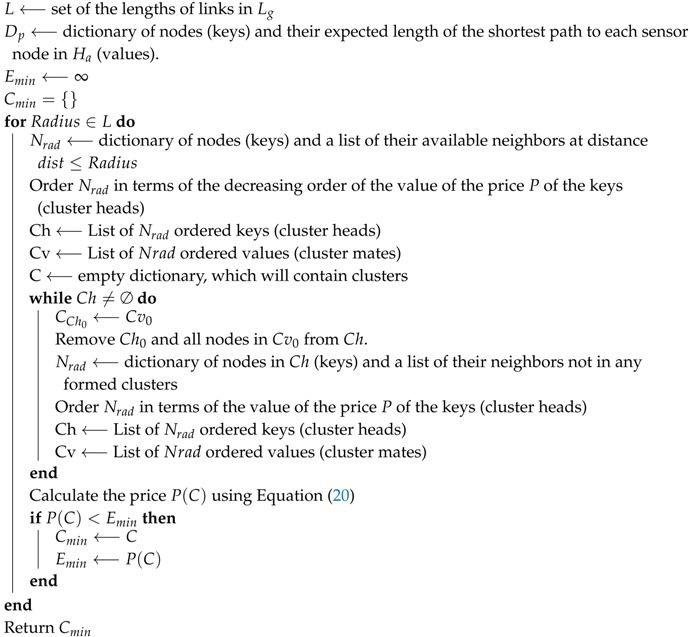



From Line 5 on, each link length (Euclidean distance between two connected nodes) is used as the clustering radius (maximum distance of nodes and cluster heads), to form a corresponding clustering *C*.

Clustering is done using a dictionary $N_{rad}$, consisting of nodes and their $H_{g}$ neighbors at a distance less than or equal to the chosen radius. Note that the radius is only chosen from a list of lengths of the $H_{g}$ links, and it is assumed to be the same for all clusters to be formed. This dictionary gets formed (Line 6), and using the pricing shown by Equation (20), it is decreasingly ordered (Line 7).

Let Ch be a list of ordered $N_{rad}$ keys (list of potential cluster heads) and Cv be the list of the corresponding keys (possible cluster members). To form the first cluster, we take the first element of the list Ch to be the cluster head, and the first list in Cv constitutes the corresponding cluster mates.

$N_{rad}$ is then updated to contain the remaining possible cluster heads and their neighbors, which are the nodes not in any of the formed clusters.

The process of using the available nodes in the list $N_{rad}$ to make one cluster is repeated (Line 11–16) until no more cluster heads are available. In this case, one clustering configuration is done, and its cost is computed using Equation (3) (Line 17).

Each new clustering-related cost is compared to the existing minimum cost to check the possibility of updating the best cluster $C_{min}$ and the corresponding cost $E_{min}$.

An example that shows graphically how Algorithm 1 works is presented below. For simplicity, only the network of type $H_{g}$ is shown. The considered cluster radius is assumed to be the maximum link length, and hence, cluster heads will be associated with all their neighbors in $H_{g}$.

**Figure 4:**
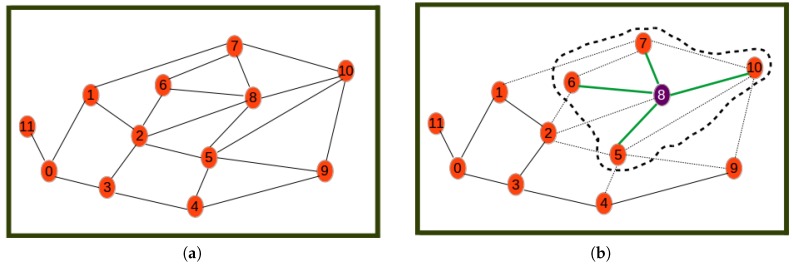
Beginning steps. (**a**) Step 0: initial network; (**b**) Step 1.

**Figure 5:**
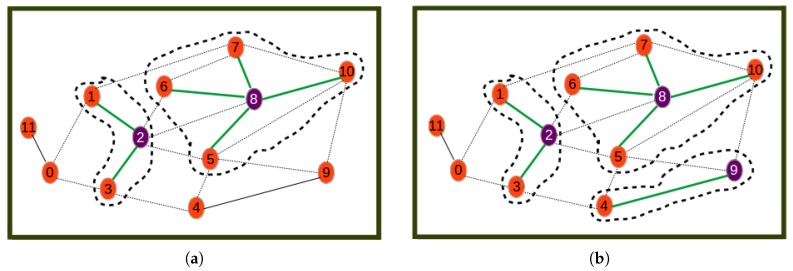
Processing steps. (**a**) Step 2; (**b**) Step 3.

**Figure 6:**
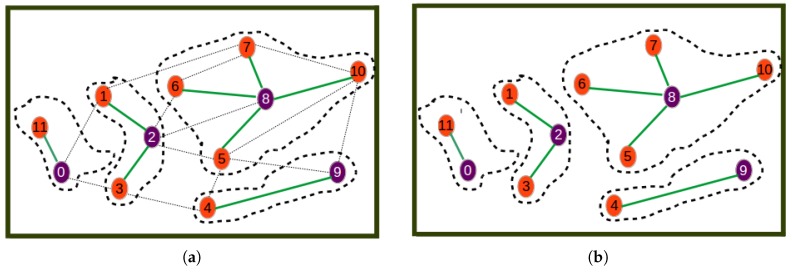
Last steps. (**a**) Step 4; (**b**) Step 5.

Figure 4, Figure 5 and Figure 6 show the different steps involved in the clustering algorithm example and are explained below:

Step 0 is the initial step revealing the initial network.
Step 1 selects Node 8 as the one with highest utility (computed by Equation (20)) to become the cluster head. The first cluster is formed by assigning all its neighbors as its cluster members.
Step 2 selects Node 2 as the next best cluster node. Here, to calculate the utility, the nodes or links involved in the formed cluster are not considered. This is why for example Node 2 has a new degree of two. The new degree of Node 0 is greater than that of Node 2 even though the utility of Node 2 is highest since it is the closest node to the remaining nodes.
Step 3 is a step where Node 9 is selected as the next best cluster head, which is joined by only Node 4 as its cluster member.
Step 4 is a step where Node 0 is selected as the last best cluster head, which is joined by Node 11 as its neighbor to form the last cluster.
Step 5 is the last phase where the resulting cluster and the corresponding communication links through which nodes have to send sensor readings to cluster heads are shown.

**Proposition** **1.** *Algorithm (1) satisfies the following properties.*

*P1.*  *It terminates.*
*P2.*  *The produced cluster heads constitute a dominating set of the network of type $H_{g}$ (see Constraint *(11b)*).*
*P3.*  *The set C of the produced clusters is a partition of the set of all nodes (see *(11c)* and *(11d)*).*

**Proof.** 

P1. **Termination property:** We first show that the algorithm terminates. Notice that the algorithm iterates over a finite set (see Line 5) and loops for some iterations (Line 11). It is sufficient to show that the inner loop on Line 11 halts. Notice that the loop starts with a finite set of nodes, and updates the set by removing at least one element during each iteration. It is clear that in at most #Ch steps, Ch=∅, which is a condition for the loop to stop.
P2. **Dominating set property:** Lines 6, 8, and 11 show that at the end, each node becomes either a cluster head or a cluster member. On the other hand, Lines 1, 2, 9, and 6 show that only neighbors of the cluster head are added in the same cluster to be cluster members. It then follows that when the algorithm halts, if a node is not a cluster head, then it is connected to the cluster head in the same cluster.
P3. **Partition property:** Line 13 shows that no node (cluster member or cluster head) belongs to more than one cluster. Hence, the formed clusters are mutually exclusive. On the other hand, Lines 6, 8, and 11 show that the algorithm halts when each node has either been a cluster member or (exclusively) a cluster head. This shows that in the end, each node belongs in a unique cluster. Hence, cluster nodes constitute a partition of the ground network nodes.

 □

## 4. Issues and Relaxation

In this section, we discuss two main issues and address them to improve the performance of the algorithm.

### 4.1. Energy Inefficiency

**Figure 7:**
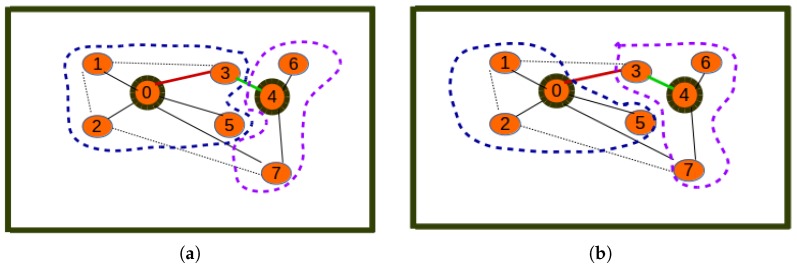
Relaxation: energy inefficiency. (**a**) Inefficient clustering; (**b**) efficient clustering.

The proposed algorithm is greedy in terms of the way cluster members are assigned to cluster heads. The assignment of all neighbor nodes (at a distance less that or equal to a threshold) to cluster heads does not necessarily lead to the best association between cluster members and cluster heads. This may lead to the case where nodes are assigned to cluster heads that are not closest to them. This would result in higher energy/cost for data aggregation on cluster heads. This issue is depicted in Figure 7a.

Figure 7a shows an inefficient clustering where Node 3 has been allocated as the cluster member of Node 0 instead of Node 4, which is the closest cluster head. Figure 7b reveals that through relaxation, nodes re-choose their corresponding clusters depending on their closest elected cluster heads, thus leading Node 3 to become a cluster member of Node 4. The solution to the energy inefficiency issue above consists of applying the relaxation algorithm below to improve the UAKM and UADC algorithms.

**Input:**
  → The graph of type $H(H_{a},H_{g})$.
  → the initial clustering (using Algorithm 1): each node and its initial cluster head denoted by *n* and $n_{ch0}$, respectively.
**Output:**
  ← A more efficient clustering.

Denote $n_{ch}$ the new cluster head of node *n*. Assume *C* is the set of all cluster heads and *N* is the set of all cluster members (all the H nodes excluding cluster heads).

### 4.2. Orphan Nodes

**Figure 8:**
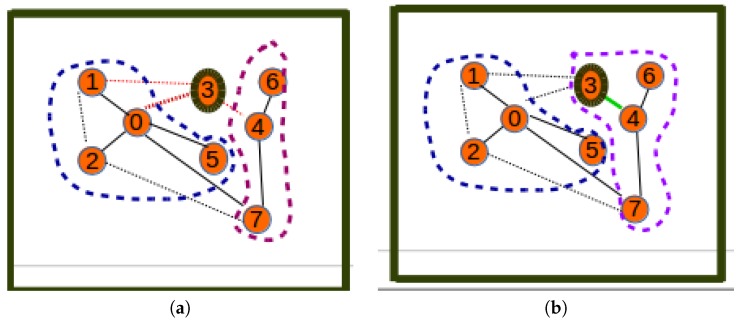
Relaxation: orphan nodes. (**a**) Inefficient network; (**b**) efficient network.

The presence of orphan nodes leading to isolated cluster heads is another issue of the proposed greedy algorithm that can reduce the utility of the hybrid network as it can lead to the UAV being tasked to collect data on a cluster-head with very reduced data. This is illustrated by Figure 8a, which reveals a sensor network with three clusters: the first cluster with Node 0 as the cluster head and Nodes 1, 2, and 5 as cluster members, the second with Node 4 as the cluster head and Nodes 6 and 7 as cluster members, and the last cluster, which has the orphan Node 3 as the isolated cluster head. By applying a distance-aware node redistribution process, the sensor network will be restructured into a two-cluster network similar to the one depicted by Figure 8b with two clusters: the first cluster with Node 0 as the cluster head and Nodes 1, 2, and 5 as cluster members and the second cluster with Node 4 as cluster head and Nodes 3, 7, and 6 as cluster members.

Figure 8a reveals a clustering where Node 3 is an orphan node in a cluster consisting of only one cluster head with no cluster member, while Figure 8b reveals a situation where the orphan cluster head is assigned to the optimal cluster whose cluster head is nearest to the orphan node, thus becoming one of its cluster members. The solution to the orphan node inefficiency issue related to the above consists of applying the cluster restructuring algorithm below to improve the UAKM and UADC algorithms. Note that while this algorithm is based on the same principles as the distance-aware node redistribution, cluster restructuring may require balancing the benefits due to energy efficiency and the data muling utility in order to decide on whether to move an orphan node into another cluster to become a cluster member or leave the orphan node in its current cluster.

**Input:**
  → The graph of type $H(H_{a},H_{g})$.
  → the initial clustering (using Algorithm 1): each node and its initial cluster head denoted by *n* and $n_{ch0}$, respectively.
**Output:**
  ← A more efficient clustering.

Denote $n_{ch}$ as the new cluster head of node *n* and ut(n) a Boolean value indicating if it is more beneficial to restructure the network. The re-clustering may be required when the cost of sending data to $n_{ch}$ is smaller than the cost of visiting the node with a UAV.

Assume *C* is the set of all cluster heads and *N* is the set of all cluster members (all the H nodes excluding cluster heads), see Algorithm 2.

**Algorithm 2:** Distance-aware cluster restructuring.

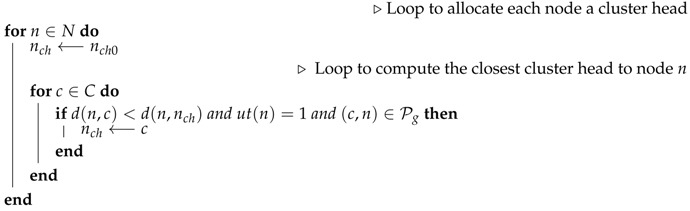



### 4.3. The Update Step

As suggested above, both the UAKM and UADC algorithms can be updated into a two-step algorithm that applies the basic algorithm first (UAKM or UADC) and thereafter balances the network using the distance-aware relaxation algorithm above. We adapt the k-means update step to achieve energy efficiency by using the fact that the knowledge of the cluster heads can help redistribute cluster members according the closeness to cluster heads, as shown in Figure 7b. The same applies to the UADC algorithm, which is complemented by a relaxation step to balance the energy consumption as suggested above.

**Remark** **1.**

- *The distance-aware relaxation algorithm proposed above may lead to energy consumption improvement.*
- *The restructuring of the terrestrial sensor network is another relaxation technique that follows the same distance-aware strategy for a different purpose, but it can also lead to energy consumption improvement.*

## 5. Results and Discussion

In this section, we report on the experimental results obtained from running the proposed algorithms in different settings. The algorithms (both UADC and relaxed UADC) are analyzed and compared to the DCC and k-means algorithms for benchmarking purposes. We considered two network topologies: (i) a random network and (ii) the city of Cape Town network used as a smart city use-case.

### 5.1. Smart City Use-Case

**Figure 9:**
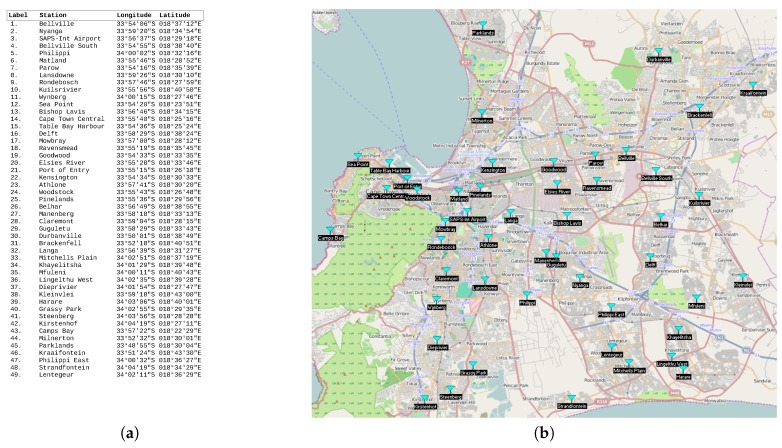
Case study. (**a**) GPS positions; (**b**) positions on the map.

**Figure 10:**
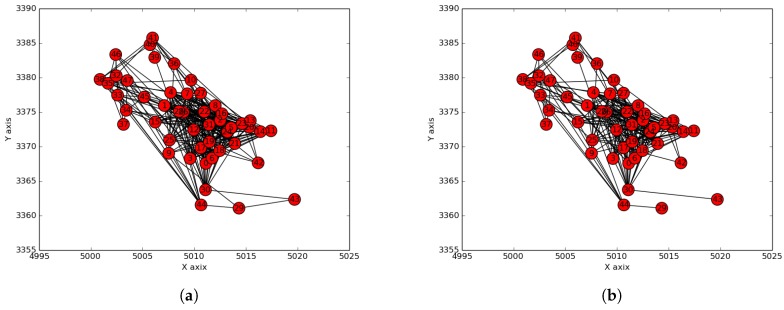
Processed networks. (**a**) Communication network considered; (**b**) UAV path network considered.

We considered the public safety network topology consisting of Cape Town (South Africa) police stations as collection points of the terrestrial/ground sensor network. This network was used as a smart city use-case aiming to provide citizen safety and city surveillance through a combination of aerial and terrestrial traffic control. The Cape Town police stations are labeled in terms of integers in the interval [1,49], and their GPS coordinates were used as their positions (see Figure 9a). The corresponding positions on a map are shown in Figure 9b. The Radio Mobile software [32] was used to create the hybrid network by having the terrestrial/ground communication network (see Figure 10a) generated using a two-step process consisting of (i) generation by the mobile radio of a terrestrial network that considers only connections whose link margin is greater than 50 dB in the white space spectrum frequency and (ii) generation by the mobile radio of an aerial network consisting of UAV paths (see Figure 10b) that considers only connections/links with a link margin between 30 and 50 dB in the same white space band.

### 5.2. Hybrid Clustering: α=10 and β≠0 and γ≠0

**Figure 11:**
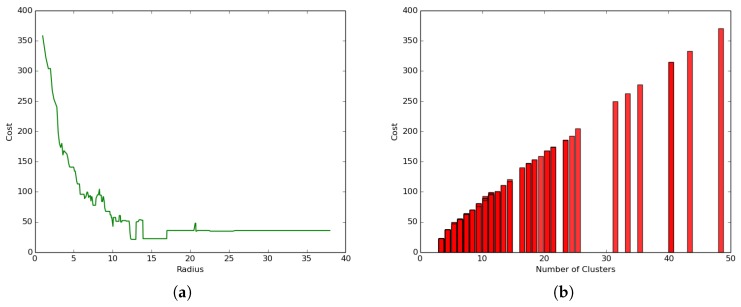
Impact of parameters on performance. (**a**) Cost versus radius; (**b**) coverage cost versus the number of clusters.

We conducted the first experiment to study the impact of the radius and number of clusters on the performance of a hybrid clustering model where both layers were considered: airborne and terrestrial network by setting the clustering parameters to α=10 and β≠0 and γ≠0. The results presented in Figure 11a show that the coverage cost (total coverage energy) reduced with the increase of the radius following a logarithmic function that led to a convergence value that did not necessarily correspond to the optimal point.

On the other hand, the results presented in Figure 11b reveal a different trend where the coverage cost (total coverage energy) increases linearly with the increase of the number of clusters. These results are in line with the one presented in Figure 11a, since a lower radius will logically lead to a higher number of clusters and subsequent higher cost, while a higher radius will logically lead to the algorithm finding a lower number of clusters and subsequent lower coverage cost resulting from a high transportation cost. The best clustering would then be the one related to the radius, which minimizes the number of clusters and hence leads to the minimum transportation cost. In Figure 11a, such an optimal radius is 13, and it corresponds to four clusters and a transportation cost of close to 25 joules.

### 5.3. Terrestrial Clustering: α=10 and β=0 and γ=0

We conducted a second experiment to evaluate the impact of the UAV presence on the hybrid clustering process by setting the parameters α=10 and β=0 and γ=0, which represent a setting where only the energy consumed for transmission and reception in the ground/terrestrial network is considered, discounting the data muling energy consumed by the UAV.

**Figure 12:**
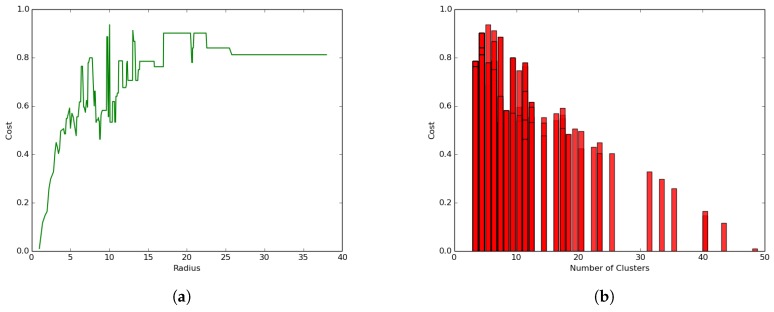
Impact of UAV on clustering. (**a**) Radius-cost; (**b**) communication network.

The results presented in Figure 12a reveal a different trend compared to the hybrid network setting in Figure 11a where:

1. The coverage cost function increases with the increase in the radius size following an exponential function leading to a convergence value where the cost becomes constant.
2. The clustering process leads to much smaller coverage cost values (less than 1.0 joule) as compared to the general case where the coverage cost values ranged between 20 and 370 joules.

Similarly, Figure 12b shows a different trend compared to the hybrid clustering in Figure 11b, where the coverage cost decreases with the increase in the number of clusters, but not necessarily following a strict linear trend. The correlation between the values in Figure 11b and Figure 12b is negative and smaller.

### 5.4. The Impact of the Cluster Head Selection Parameter on Performance

We conducted a set of experiments to evaluate the impact of the cluster head selection policy on performance by setting the parameters ψ=100 and varying λ from 0 to 1 as follows

- λ=0 expressing a distance awareness policy.
- λ=0.25 expressing a balanced policy with a more focused distance awareness trend.
- λ=0.5 expressing a fair, balanced policy between density and distance awareness.
- λ=0.75 expressing a balanced policy with a more focused density awareness trend.
- λ=1 expressing the density awareness policy.

The goal was to assess how the three different policies would impact the overall coverage cost.

**Figure 13:**
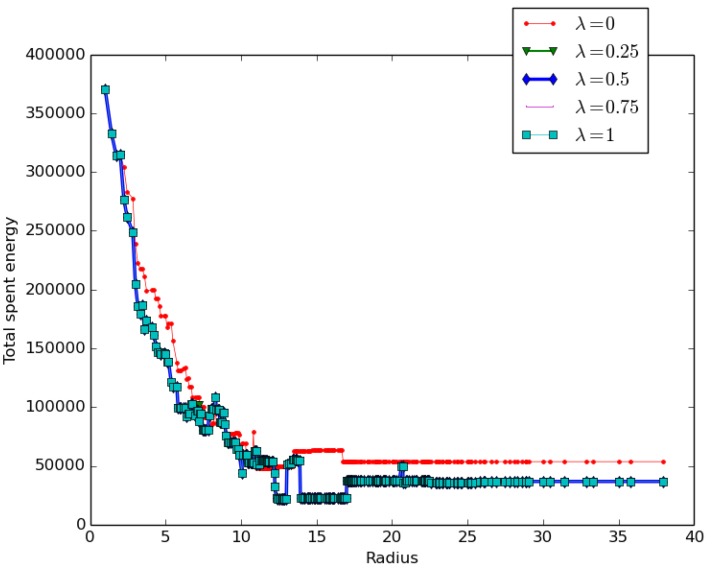
Impact of the cluster-head selection policy.

The results presented in Figure 13 revealed that:

- Distance awareness decreases the total coverage cost more slowly than density awareness: at any given radius, the distance awareness policy cost is higher than the density awareness policy cost, as revealed by the red curve corresponding to λ=0.
- Any balanced policy 0<λ<1 leads to the same and lower energy cost as the density awareness policy λ=1.

### 5.5. UADC versus DCC Performance Comparison

**Figure 14:**
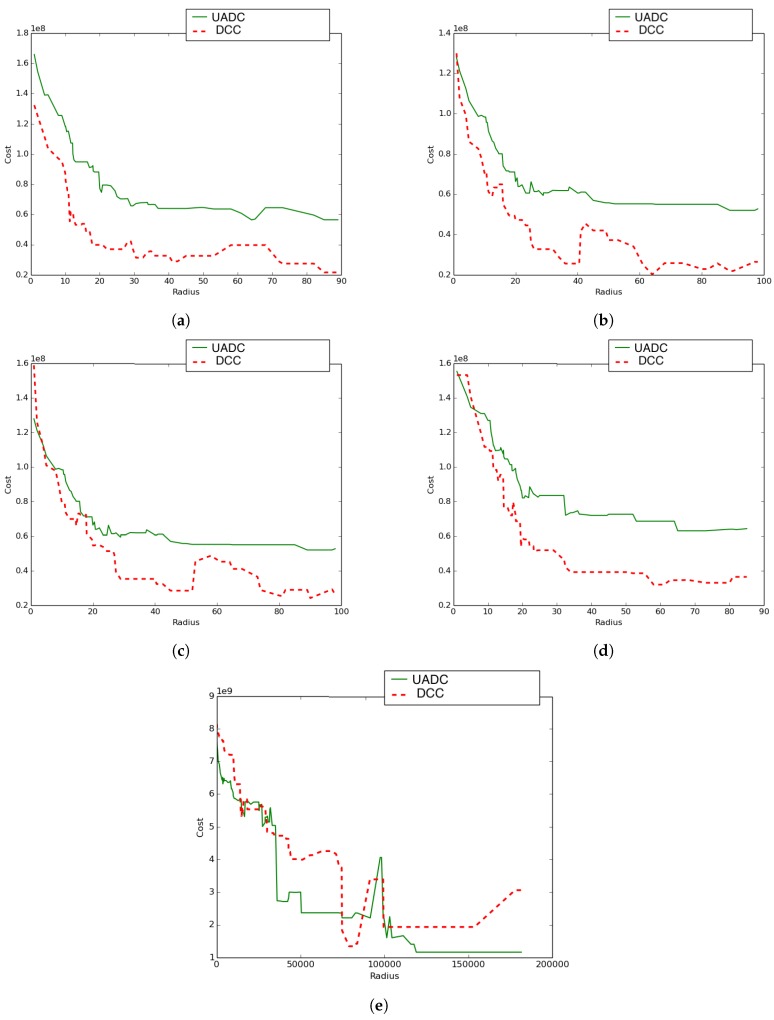
Algorithms comparison on different topologies. (**a**) Case 1; (**b**) Case 2; (**c**) Case 3; (**d**) Case 4; (**e**) Case 5. UADC, UAV-Aware DCC.

We conducted another experiment to compare the performance of the DCC algorithm (using only density awareness: λ≠0 and ψ=0) to the UADC algorithm (using both density and distance awareness: λ≠0 and ψ≠0) using a variety of network topologies. The following five settings/cases (see Figure 14) were considered:

- Case 1: The UAV’s paths constitute a proper sub-network of the terrestrial communication network.
- Case 2: The terrestrial communication network is a proper sub-network of the UAVs’ network. This has been achieved by interchanging the networks chosen for the experiment in Figure 14a.
- Case 3: The two networks (terrestrial and aerial) are the same. Here, the assumed network is shown by Figure 10a.
- Case 4: In this experiment, positions were kept the same, and for both types of networks, the connections were generated randomly.
- Case 5: In this experiment, both the positions and links of both networks were generated randomly. The total number of considered nodes was still 48, and the positions were generated by randomly selecting the coordinates from a normal distribution with mean = 500 and a standard deviation of 300 (N(500,300)).

**Figure 15:**
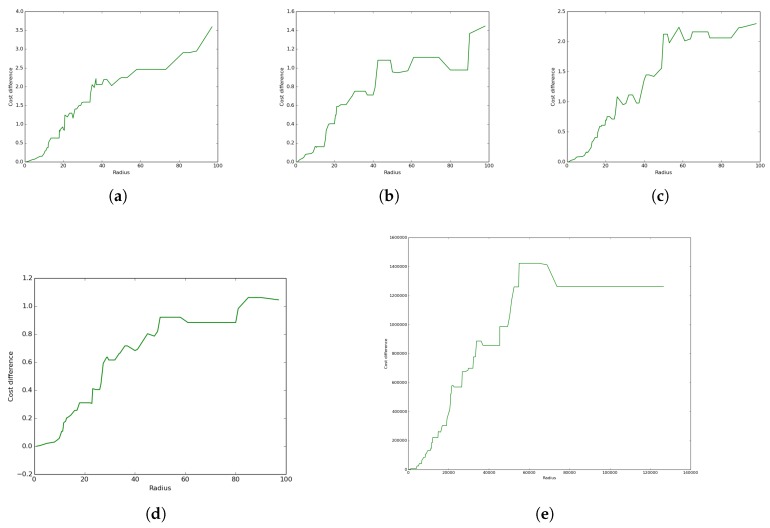
Algorithms difference based on different topologies. (**a**) Case 1; (**b**) Case 2; (**c**) Case 3; (**d**) Case 4; (**e**) Case 5.

Figure 15 reveals the total difference in coverage cost between the UADC and DCC algorithms as a function of the radius. These results reveal that:

- With the exception of Case 5, UADC leads to higher coverage cost compared to DCC as a result of the data muling cost due to the energy consumption of the UAV.
- The case where the terrestrial communication network is a proper sub-network of the aerial network (Case 2) leads to lower coverage cost compared to the reverse case (Case 1) where the aerial network is a sub-network of the terrestrial network.
- The lowest UADC cost is achieved when the aerial network and the terrestrial networks are the same (Case 3).
- The case where both networks have the same positions, but randomly-generated connections (Case 4) leads to higher coverage cost compared to the case where both networks are the same (Case 3) for both the UADC and DCC algorithms.
- The case where positions and links are randomly generated for both networks (Case 5) is the only case where the UADC algorithm outperforms the DCC algorithm for some of the higher radius sizes.

The proposed algorithm evolves. It shows that when the UAVs’ paths constitute a sub-network of the communication network, the adoption of the DCC’s policy was best for all the algorithm’s steps (see Figure 15a). However, considering the converse case (Figure 15b), the UAV-aware policy outperformed the adopted DCC in only two cases, but the lowest energy corresponded to the adoption of the DCC. For the case in Figure 15d, we observe more cases where the UAV-aware policy was better than adopting the DCC, but still, the minimum energy corresponds to the DCC adoption. Randomly generating the nodes positions, Figure 15e shows that the UAV-aware policy was the one corresponding to the lowest energy and hence outperformed the adoption of the DCC.

### 5.6. The Impact of Relaxation on Performance

In this subsection, we show the impact of the relaxation algorithm (see Algorithm 3) on performance by revealing the difference of the total coverage cost between the UADC algorithm without and with relaxation for the same five different cases described above.

**Algorithm 3:** Distance-aware node redistribution.

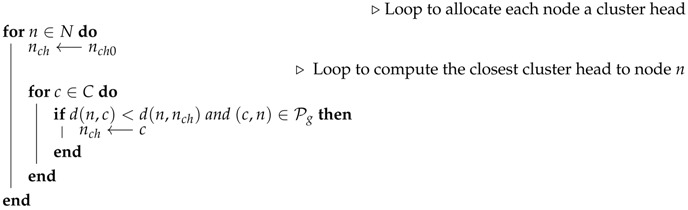



The results presented in Figure 15 for all five cases reveal positive values for all radii. This reveals that the proposed distance-aware relaxation (and similarly, the distance-aware restructuring) had a positive impact on the performance achieved by the UADC algorithm. Furthermore, the figures reveal an increase of the coverage cost difference with the radius. The results also reveal a variation of such an increase with the cases where it is more pronounced for some cases compared to others, as shown by the slope and values of the different cost difference functions.

### 5.7. Reliability of the Family of k-Means Algorithms

**Figure 16:**
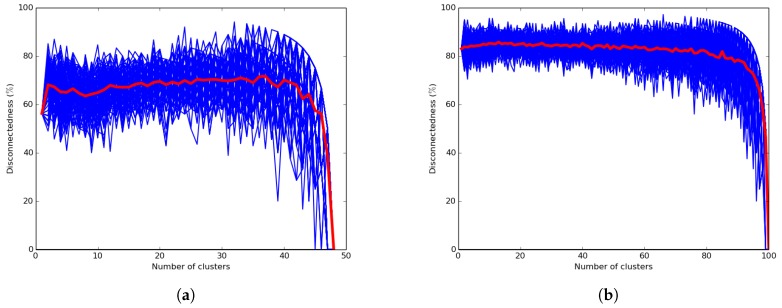
K-means algorithm. (**a**) Average disconnectedness: Cape Town network; (**b**) average disconnectedness: random network.

In this subsection, we evaluate the connectedness of the network configuration, which expresses the reliability of the k-means and UAKM algorithms in terms of intra-cluster connectivity. The connectedness is a key property that determines the efficiency of the data muling process handled by the UAV in the hybrid network scenario since the sensor readings are collected by the moving UAV only when visiting cluster heads. Therefore, a highly-disconnected network will lead to high missing data. Note that a poorly-connected and less reliable network configuration will reveal lower intra-cluster connectivity, while a more reliable and highly-connected network configuration will result in higher intra-cluster connectivity. The results are shown in Figure 16a,b and in Table 2 in terms of average disconnectedness. Figure 16a and Table 2 reveal the results for the city of Cape Town network depicted by Figure 10a, while Figure 16b shows the results of a random network. The average disconnectedness was computed as the percentage of (orphan) nodes that have been assigned to clusters by the k-means algorithm, but that were not connected to related cluster heads. A hundred runs were performed for every run and every value *k* of the cluster with the number *k* of clusters ranging from one to the total number of nodes of the network. Figure 16b shows the average disconnectedness for a random 100-node network where the coordinates of the 100 nodes’ positions were randomly chosen from a standard normal distribution of size 1000, and the links were also randomly generated to get a connected graph.

On the other hand, Table 2 reports on the disconnectedness results of the UAKM algorithm where the value *k* was set to four to reflect the optimal number of clusters. For every cluster, the colored and bold nodes in Table 2 are the ones that are disconnected from their corresponding cluster heads.

The results presented in Figure 16 for the k-means algorithm show that the expected cluster’s disconnectedness was significantly high. They also show that it was close to zero only when all cluster heads were orphan nodes. This could lead to excessive energy consumption resulting from the UAV visiting each and every node of the network for data muling. Figure 16b shows that the disconnectedness level of the random network was higher than the Cape Town network disconnectedness. Furthermore, the results presented in Table 2 for the UAKM algorithm also show significant disconnectedness in each of the four clusters. This confirms that the *k*-means algorithms were significantly less reliable than the proposed UADC algorithm.

## 6. Conclusions

### 6.1. Summary

In this paper, a model for optimal sensor network design has been provided where a multi-sink ground-based terrestrial sensor network is expanded by an airborne network using a UAV to ferry the sensor data from the sinks of the terrestrial sensor network to the gateway where the data have to be processed. The coverage problem has been mathematically formulated as an optimization problem aiming at finding the optimal number of clusters to achieve an energy-efficient hybrid terrestrial/airborne sensor network using an UAV as the mobile gateway. A clustering model has been proposed and discussed to address the defined problem. It has been shown that the energy spent by the UAV data muling has a big impact on the change in the energy consumed by the whole process of data transport. The efficiency of the proposed model has been compared with DCC and the k-means algorithms, and the results showed that it is more reliable.

### 6.2. Future Work

This work has been proposed as part of the Internet-of-Things in Motion, a project that targets both data muling/ferrying using a team of UAVs working in a coalesced manner such as in [33,34], or independently based on a competitive model as suggested in [35], or a collaborative model as proposed by [36,37]. The integration of the proposed networking engineering model to enhance service differentiation in complex sensor networking scenarios with mixed devices as suggested in [38] by balancing sensor roles and UAV proximity is another avenue for future work. The model is also currently being integrated into the smart parking model presented in [39] with service differentiation for ground sensor networks as suggested earlier. The work proposed in this paper can also be used in the future to complement the work done in [40] as network engineering that considers a hierarchical topology as opposed to the flat topology suggested earlier with the expectation of reducing OPEX and CAPEX. Supporting food security through drought mitigation as suggested in [41,42] is another technique that, in future research work, can benefit from the network engineering principles proposed in this paper by using UAVs as airborne cameras and data mules capable of ferrying agricultural data from fields to processing places where machine learning algorithms are applied to improve precision agriculture.

Distance-based relaxation was used in this paper as a way of mitigating issues related to the energy inefficiency and orphan node issues of the heuristic clustering algorithm. The redistribution of cluster members to achieve a more balanced network is another relaxation technique that can be applied to the two clustering algorithms studied in this paper for energy efficiency and the avoidance of orphan nodes (cluster members with no cluster or cluster heads with no cluster members). A combination of distance awareness and cluster members’ redistribution is a third technique that can also be applied to the two clustering algorithms. The design and implementation of these techniques is another avenue for future research work.

## Figures and Tables

**Table 1:** Parameters and their corresponding values.

Parameter	Network 1	Network 2	Network 3	Units
n	100	100	200	
$E_{e}$	20	20	20	nJ/bit
$E_{p}$	21	21	21	nJ/bit/signal
$e_{f}$	1	1	1	pJ/bit/m^2^
*L*	30	60	30	m
*a*	0.0008	0.0008	0.0008	
*b*	9	9	9	
*D*	6	6	6	
$\bar{E}+\bar{e}$	3	3	3	nJ/bit/signal

**Table 2:** Average disconnectedness: UAKM algorithm.

Cluster Head	Cluster Members	Disconnectedness
33	38, 47, 37, 35, 34, 32	66.6
3	25, 45, 15, 17, 44, 30, 29, 0, 6, 9	80.0
2	42, 24, 26, 27, 20, 21, 22, 23, 28, 43, 1, 5, 4, 7, 8, 11, 13, 12, 14, 16, 19, 18, 31	52.2
36	46, 10, 39, 40, 41	80.0
